# Complex tone stimulation may induce binaural diplacusis with low-tone hearing loss

**DOI:** 10.1371/journal.pone.0210939

**Published:** 2019-01-25

**Authors:** Issei Ichimiya, Hiroko Ichimiya

**Affiliations:** Ichimiya Clinic, Kitsuki City, Oita, Japan; University College London, UNITED KINGDOM

## Abstract

To clarify the possible mechanism causing binaural diplacusis with low-tone hearing loss, two psychoacoustic experiments were performed with 20 healthy subjects, using harmonic complex tones. In the first experiment, two tones were presented unilaterally, either from the right or left side. One of the tones presented was higher in frequency in terms of the fundamental component, but lower or equal in frequency in terms of the highest component, than the other tone. The subjects were asked which tone was higher in pitch after listening to both tones. They were also asked to compare tones in which low-tone components were eliminated. In the second experiment, the subjects heard these complex tones binaurally, with low-tone components eliminated in one ear. In the first experiment, most subjects perceived pitch direction, that is, higher or lower, in a reverse way when low-tone components were eliminated from the complex tones. In the second experiment, approximately half of all subjects heard the tones at different pitches in both ears. Under certain conditions, complex tone stimulation may induce binaural diplacusis when low-tone hearing is lost in one ear.

## Introduction

Binaural diplacusis is defined as the phenomenon of hearing the same tone at different pitches in both ears. Findings reported in the literature suggest that this phenomenon is more prevalent in individuals with asymmetric hearing loss [1]. For the assessment of diplacusis, pitch-matching tests have been conducted and indicate that the perceived pitch in the impaired ear shifts to the higher-frequency side, although this trend was not typically observed in patients with low-tone hearing loss [2,3]. The differences between types of hearing loss seem to be explained by different mechanisms of pitch perception according to frequency.

Approximately half of all patients with Meniere's disease, whose representative audiological symptom is low-tone hearing loss, complain of binaural diplacusis [4]. Studies indicate that the affected ear can perceive lower, higher, or equal pitch compared to the opposite, unaffected ear [3,5]. These subjective variations in pitch are generally explained in terms of different stages of the disease.

It must be noted that the above-mentioned studies were conducted using pure tones. However, many of the sounds encountered in everyday life, such as those produced by musical instruments and certain speech sounds, are harmonic complex tones [6]. A harmonic complex tone is composed of a number of pure tones, each of which has a frequency that is an integer multiple of the frequency of a common fundamental component. For example, a note of A3 played on the piano has a fundamental component with a frequency of 220 Hz, a second harmonic with a frequency of 440 Hz, a third harmonic with a frequency of 660 Hz, etc. One should not dismiss the possibility that patients are aware of binaural diplacusis or pitch shift when they hear these complex tones.

For a pure tone, pitch perception corresponds to the frequency, while for a harmonic complex tone, it corresponds to the fundamental frequency [6]. We hypothesized that such a difference in pitch perception for a complex tone may also explain binaural diplacusis or the pitch shift in addition to the speculations by the previous studies in which pure tones were used.

In this study, we enrolled normally hearing subjects to demonstrate how pitch perception of a complex tone can be shifted when low-tone components are eliminated. We speculated that under certain conditions, complex tone stimulation may induce binaural diplacusis.

## Materials and methods

### Subjects

Twenty subjects (10 male and 10 female subjects, with a mean age of 40.6 years, standard deviation = ±10.3) were tested. It was confirmed that all 20 subjects, who reported normal hearing and no neurological conditions, were able to hear all the tone components that were used in the experiments. The study protocol was reviewed and approved by the clinical research ethics committee of Ichimiya Clinic. Written informed consent was obtained from all subjects prior to the study.

### Tone preparation and equipment

The stimulation tones were prepared using a publicly available software provided by Dr. Y. Nakajima (Kyushu University, Fukuoka, Japan); each tone had a 500-ms duration and rise and fall times of 10 ms. All tones were saved in the form of WAV files (16 bits/44.1 kHz).

**Fig 1:**
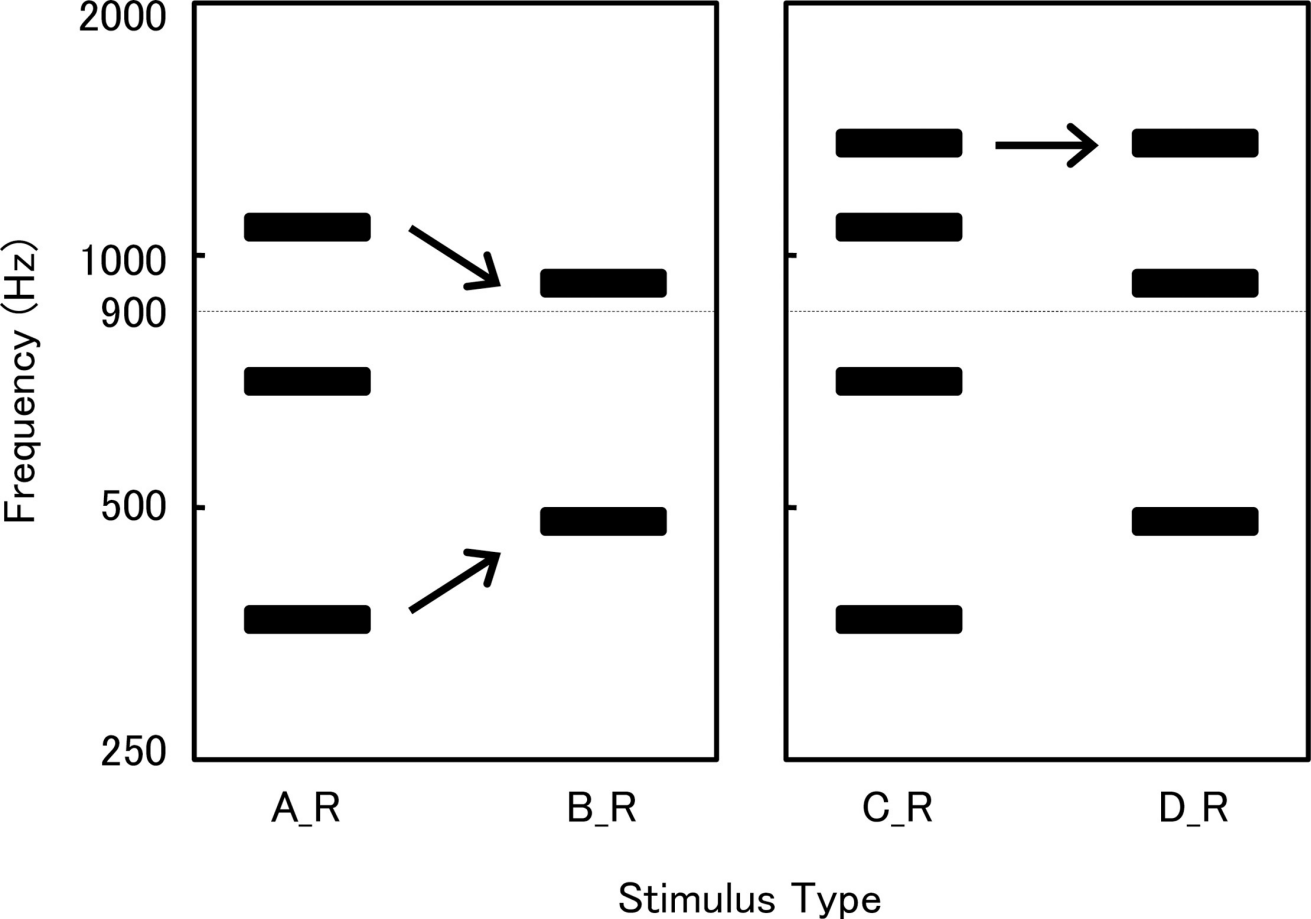
Schema of the tones for the right ear test. The fundamental component of (B_R) is higher in frequency than that of (A_R). The highest component of (B_R) is lower in frequency than that of (A_R). Tones (C_R) and (D_R) are identical to (A_R) and (B_R), respectively, except both (C_R) and (D_R) contain one higher component of equal frequency. Arrows indicate the differences in frequencies between the tones.

**Fig 2:**
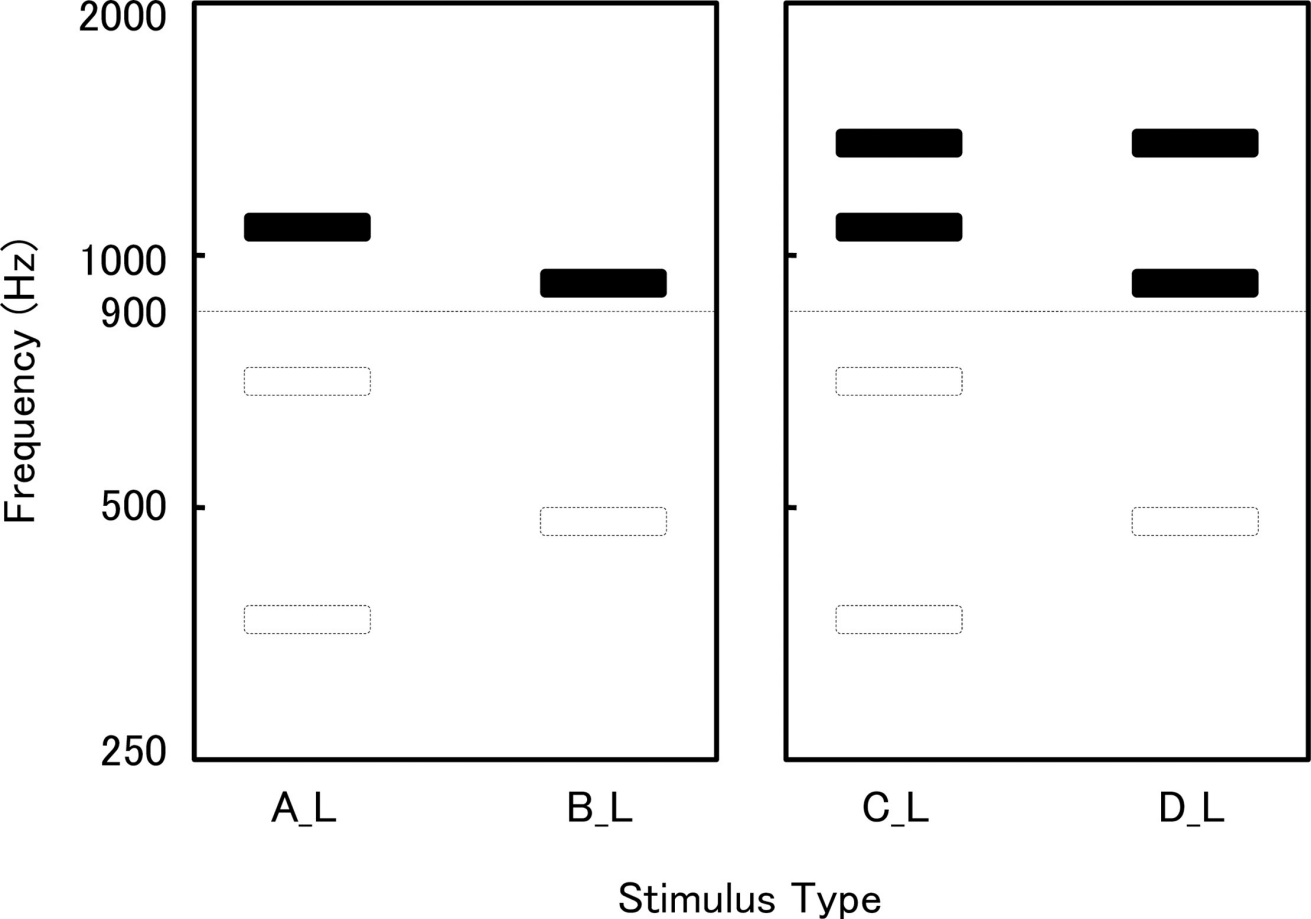
Schema of the tones for the left ear test. The tones for the left ear test are named (A_L), (B_L), (C_L), and (D_L). These are complex or pure tones whose components of less than 900 Hz were eliminated, in addition to the tones of the right channel.

Figs 1 and 2 show the schema of the stimulation tones. The harmonic complex tones (A) and (B) were devised as follows: (1) the fundamental component of (B) was higher in frequency than that of (A); (2) the highest component of (B) was lower in frequency than that of (A); (3) both (A) and (B) contained one component whose frequency was higher than 900 Hz. The harmonic complex tones (C) and (D) were identical to (A) and (B), respectively, except that both (C) and (D) contained one higher component of equal frequency. The concrete frequencies of the components were, (A): 339, 678, 1017 Hz, (B): 452, 904 Hz, (C): 339, 678, 1017, 1356 Hz, (D): 452, 904, 1356 Hz. All components were mixed at the same sound pressure level (i.e., 70 dB sound pressure level). For the right ear test, tones of the left channel were eliminated. These tones were named (A_R), (B_R), (C_R), and (D_R). Tones for the left ear test were named (A_L), (B_L), (C_L), and (D_L). These were complex or pure tones whose components of less than 900 Hz were eliminated, in addition to the tones of the right channel. This denotes that the left ears were selected for simulating low-tone hearing loss for the experiments described below.

Using an Aspire S3 computer (Acer America Corporation, San Jose, CA, USA) with a USB audio processor (SE-U55SXII; Onkyo Digital Solutions, Tokyo, Japan), auditory stimuli were delivered through dynamic headphones (MDR-7506; Sony, Tokyo, Japan). Prior to all the experiments, the subjects were asked if the test tones they heard monaurally in each ear were "too low," "comfortable," or "too loud." We had arranged to adjust the level of the tones to “comfortable” if the subjects reported that they were "too low" or "too loud." However, because all subjects responded that the level was "comfortable" for all tones, no change was required. They received written instructions via the computer monitor and were asked to respond to the questionnaire in writing.

### Experimental procedure

In the first experiment, the computer monitor showed two buttons that played the stimulation tones unilaterally, either from the right or from the left side. Subjects were asked to compare these tones by clicking the buttons and to decide which tone was higher in pitch. They were allowed to click the buttons multiple times before making a decision. The tone pairs to compare were (A_R) vs. (B_R), (A_L) vs. (B_L), (C_R) vs. (D_R), and (C_L) vs. (D_L). The subjects’ right and left ears were tested in turns, but the tone pairs and the order of the two buttons were presented randomly. Each task was repeated twice. When the results of the same task differed, they were considered ambiguous.

**Fig 3:**
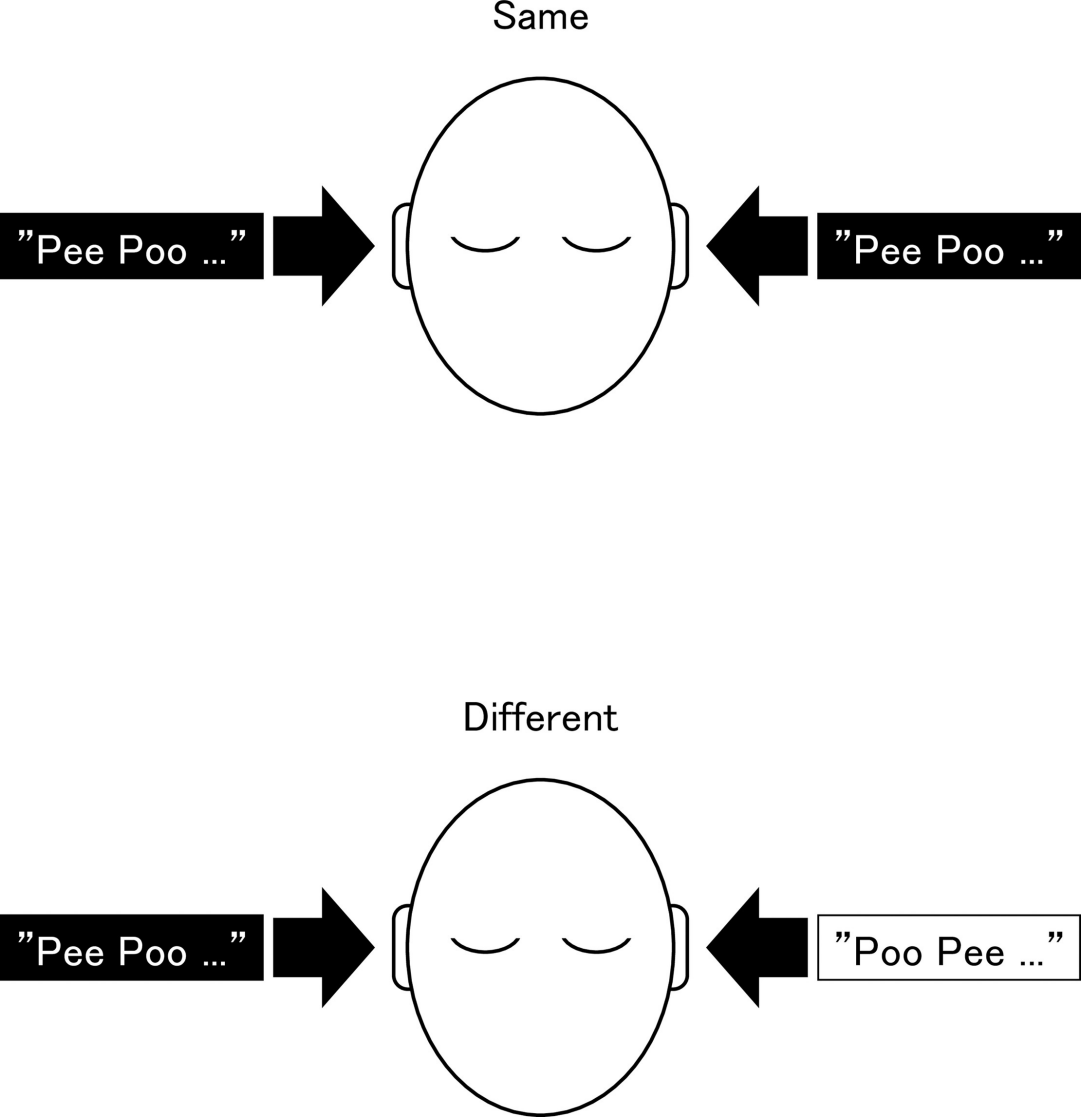
Illustration briefly describing the second experiment. Subjects were asked whether they had heard the sequence of the tones in the left ear at the same pitches or at different pitches compared to those in the right ear. Before obtaining the response, this illustration was shown to them for a better comprehension of the task.

In the second experiment, subjects heard the sequence of tones in both ears simultaneously. They heard the tones (A_R) plus (A_L), followed by (B_R) plus (B_L). The tones were repeated with a 500-ms silent interval. Because the tones used were the same as those used in the first experiment, each tone component in both ears was at the same sound pressure level. However, the overall levels of the stimuli in the left ear were lower than those in the right ear as components less than 900 Hz were eliminated for the left ear. This experimental condition simulates unilateral low-tone hearing loss. After listening, subjects were asked whether they had heard the sequence of the tones in the left ear at the same pitches or at different pitches as that in the right ear. Before obtaining the response from the subjects, an illustration (Fig 3) was shown to them for a better comprehension of the task. The same task was also conducted using the tones (C_R) plus (C_L), followed by (D_R) plus (D_L).

### Statistical analysis

Fisher's exact probability test and the chi-square goodness-of-fit test were used for statistical analysis. A p-value of < 0.01 was considered to be statistically significant.

## Results

**Fig 4:**
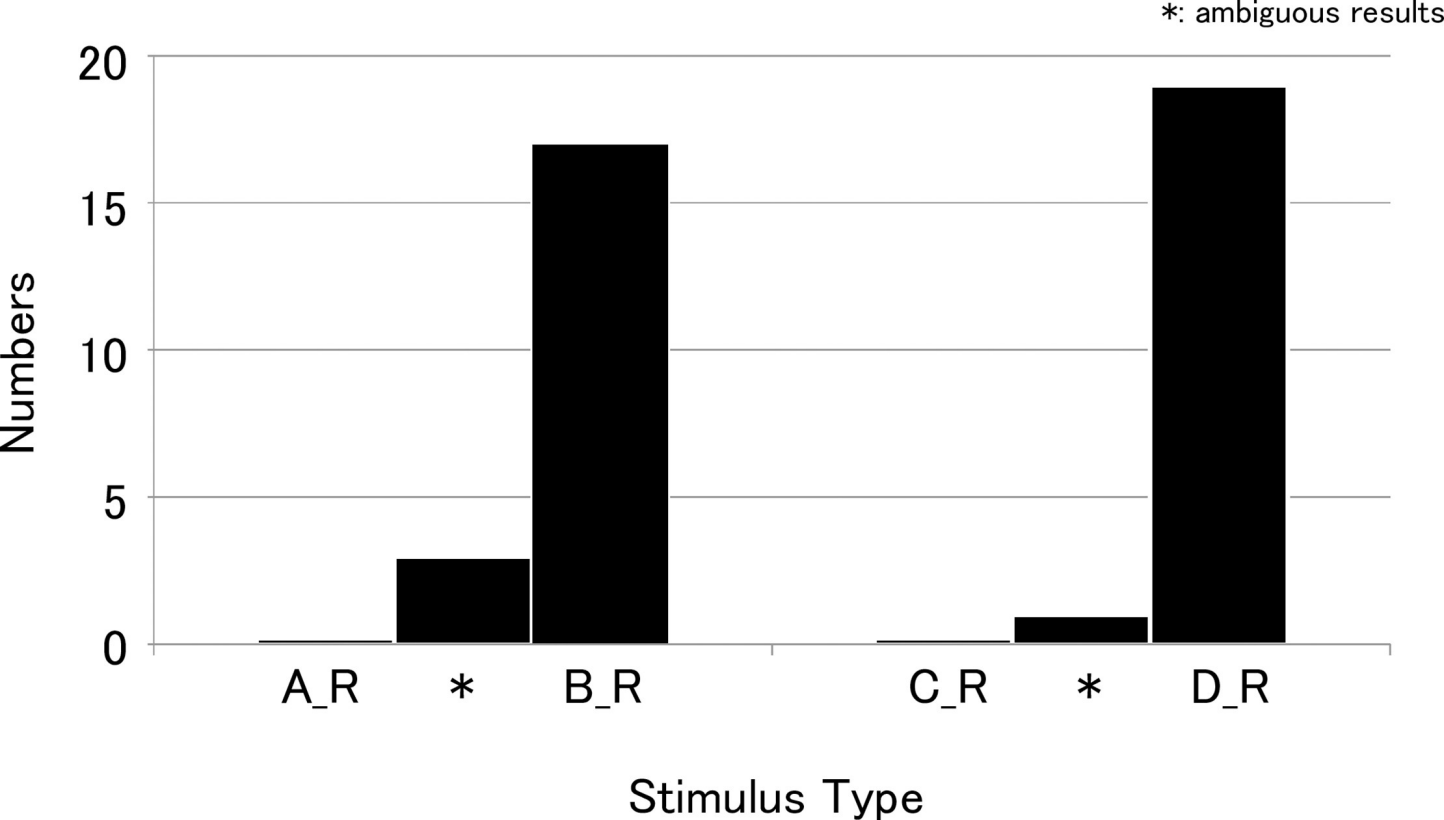
Results of the first experiment for the right ear test. When complex tones (A_R) and (B_R) were compared in the right ear, most subjects judged (B_R) as higher in pitch. Similar results were obtained in the additional experiment in which complex tones (C_R) and (D_R) were used. Most subjects judged (D_R) to be higher in pitch. *: subjects showed ambiguous results.

**Fig 5:**
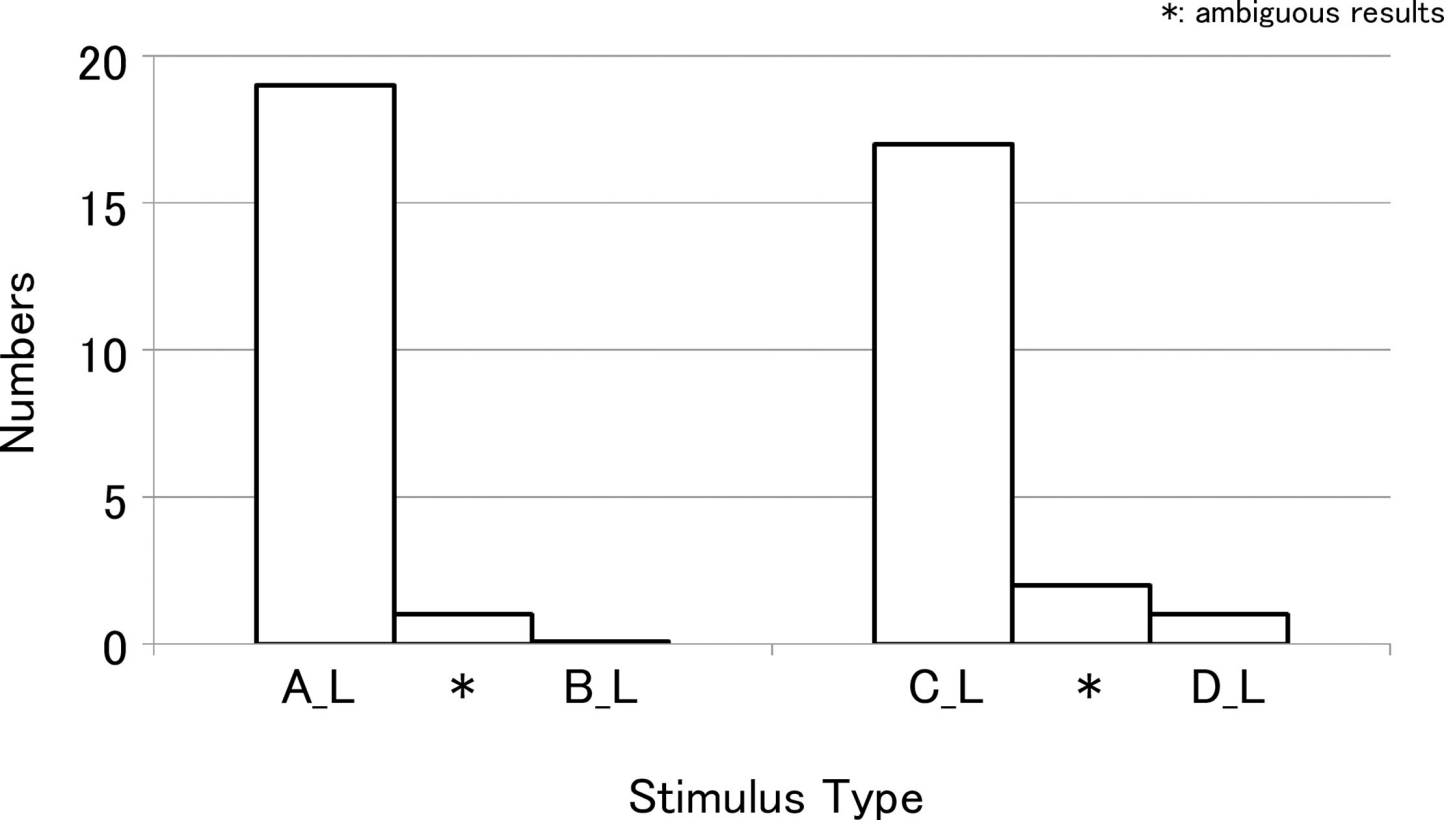
Results of the first experiment for the left ear test. In the left ear, which simulated low-tone hearing loss, (A_L) or (C_L) was judged higher in pitch by most subjects (Fisher's exact probability test, p < 0.01). There was one subject that judged (D_L) as higher in pitch than (C_L). *: subjects showed ambiguous results.

Figs 4 and 5 show the results of the first experiment. (A_R), (B_R), (C_R), and (D_R) indicate the harmonic stimuli presented to the right ear including the lower harmonic components, and (A_L), (B_L), (C_L), and (D_L) indicate the harmonic stimuli presented to the left ear excluding the lower harmonic components. When the complex tones (A_R) and (B_R) were compared in the right ear, most subjects judged (B_R) to be higher in pitch. In the left ear, (A_L) was judged higher in pitch by most subjects. This tendency was statistically significant (Fisher's exact probability test, p < 0.01). Some subjects showed ambiguous results, but no subject showed a response pattern opposite to the overall pattern, that is, (A_R) higher in pitch in the right ear test and (B_L) higher in pitch in the left ear test.

Similar results were obtained in the additional experiment in which complex tones (C) and (D) were used. In the right ear, most subjects judged (D_R) to be higher in pitch. In the left ear, (C_L) was judged higher in pitch by most subjects (Fisher's exact probability test, p < 0.01). Not only were there some subjects with ambiguous results, but there was also one subject that judged (D_L) to be higher in pitch in the left ear test.

**Fig 6:**
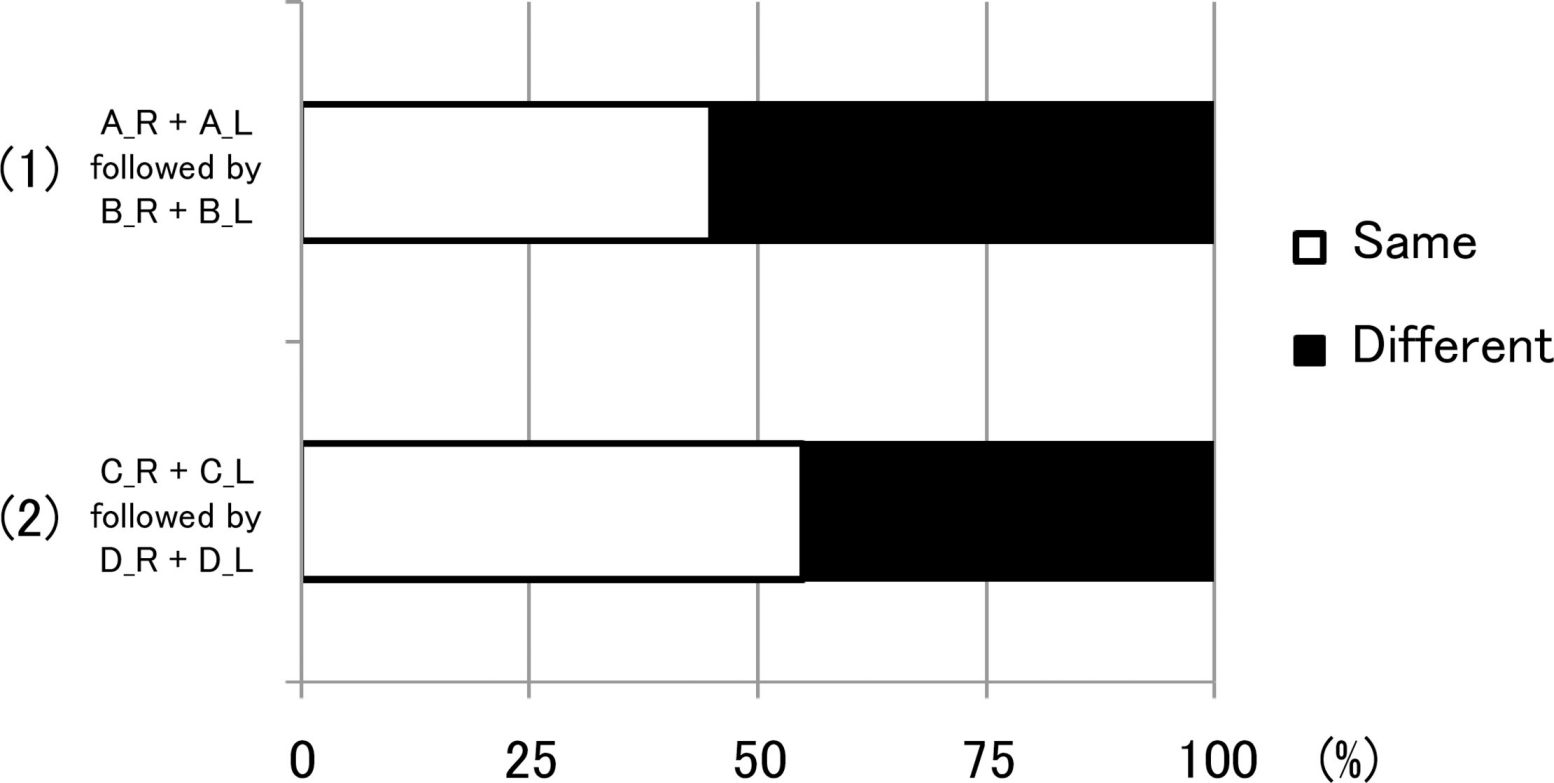
Results of the second experiment. The results of the second experiment in which subjects heard the sequence of tones in both ears simultaneously. (1) When they heard the tones (A_R) plus (A_L), followed by (B_R) plus (B_L), approximately half of the subjects heard the sequence of the tones in the left ear at different pitches from that in the right ear. (2) When they heard the tones (C_R) plus (C_L), followed by (D_R) plus (D_L), the results were similar to those of (1).

The results of the second experiment are shown in Fig 6. The results of the two tasks were similar. Approximately half of the subjects heard the tones at different pitches in both ears when they heard the sequence of tones binaurally. However, there were no statistical differences between the number of subjects that heard the tones at same pitches and the number of subjects that heard the tones at different pitches (chi-square goodness-of-fit test).

## Discussion

The results of the present study were not surprising. However, we believe that the findings of this study are valuable because, to our knowledge, no previous reports have associated complex tones to pitch shift caused by low-tone hearing loss. What we would like to emphasize here is that previous studies using pure tones could only partially reveal pitch shift caused by hearing loss. When it comes to the evaluation of pitch shift and diplacusis, one should pay attention to the features of complex tones that we always hear in our everyday life.

In the first experiment of the present study, we demonstrated how monaural pitch perception of complex (or pure) tones can be shifted by low-tone hearing loss. In our previous paper [7], psychoacoustic tasks for assessing pitch discrimination thresholds were performed with normal subjects. In that study, we found that the ability to judge the pitch of two pure tones as higher or lower varies considerably among subjects compared to the ability to detect the pitch pattern of the consecutive tones. Based on that data, the differences in tone frequencies in the present study were set above the detection thresholds. When pitch reception is analyzed, one must also be aware of the "order effect" that may affect judgment [8]; when subjects hear, for example, Tone A, followed by Tone B, and are asked to judge whether the pitches rise or fall, the order of the stimulus pair (AB or BA) may lead to different results. To avoid this effect, in our study, we showed on the computer monitor two buttons whose order was presented randomly and allowed the subjects to hear the test tones multiple times before making a decision. We therefore assume that the subjects in this study judged the pitch as higher or lower more accurately. This is supported by the results showing that all subjects except one judged pure tones that were higher in frequency as higher in pitch (i.e., (A_L) vs. (B_L)).

The fundamental component of complex tone (B) was higher in frequency than that of (A). This is probably the reason why most subjects perceived (B_R) as higher in pitch than (A_R) in the right ear test. Regarding the left ear test, (A_L) and (B_L) were pure tones when components less than 900 Hz were eliminated. Between these two pure tones, (A_L) was the one higher in frequency. It is thus quite reasonable that most subjects judged (A_L) as higher in pitch in the left ear test. The above results indicate that most subjects perceived pitch direction in the monaural condition, that is, higher or lower, in a reverse way when low tones were eliminated in these complex tones.

Prior to the present experiment, we had speculated that the comparison of complex tones (A) and (B) would lead to ambiguous results because both (A) and (B) contained a small number of components and because these tones differed in opposite directions regarding the frequency of the fundamental component (A < B) and of the highest component (A > B). We thus prepared an additional experiment in which the tones (C) and (D) were used. Both (C) and (D) had one more component, and the highest components of (C) and (D) were devised to be identical. Unexpectedly, there were only a few subjects who showed ambiguous results in the first experiment using (A) and (B), so that the results of the additional experiment only served as confirmation. Unlike (A) and (B), however, (C) and (D) were still complex tones, after their low-tone components were eliminated. The finding that most subjects judged (C_L) as higher in pitch in the left ear test suggests that most subjects based their monaural pitch perception on the average spectral frequency. There was, however, one subject that judged (D_L) as higher than (C_L). In this case, the pitch might have been perceived as the missing fundamental frequency [6].

In the second experiment we simulated how patients with low-tone hearing loss hear complex tones in both ears. Assuming that the sound source is in front of the patient, the stimuli were of the same level between the two ears, with the exception of eliminating the low-tone components on the left side. This signifies that the assumed patients' low tone hearing threshold was above the sound pressure level that was presented. In our preliminary experiment, one pair of tones was presented and subjects were asked if they heard the tones at different pitches in both ears. However, probably because low-tone elimination changes the timbre of complex tones, the subject may have been confused, and that experiment failed to yield results. Thus, we considered that we needed to devise some method to attract more attention to pitch and to distract attention from timbre. Hence, in the second experiment, we repeatedly presented the two tone pairs in that the subjects would perceive pitch direction in a reverse way in both ears and asked whether they had heard the sequence of the tones at same pitches or at different pitches. As a result, approximately half of the subjects claimed that they heard the tones at different pitches in both ears. Considering this result in connection with the result of the first experiment, it is speculated that pitch shift caused by the elimination of low-tone components might have caused hearing the tones at different pitches in both ears, which appears to correspond to binaural diplacusis. However, because we cannot exclude the possibility that some subjects have mistaken differences in timbre for differences in pitch and because the latter half of the subjects heard the tones at same pitches in both ears, the result of the second experiment does not suffice to support the notion that complex tone stimulation can induce binaural diplacusis in this experimental condition. The pitch shift by excluding the lower harmonic components may not be the main cause of binaural diplacusis. However, the results of the first experiment can explain, under certain conditions, a possible mechanism that underlies bilateral diplacusis caused by complex tones. When patients cannot hear the tone sequences from the sound source simultaneously in both the ears, they will perceive the pitch in the impaired ear to be different from that in the normal ear, if they hear the same tone sequences consecutively in one ear and then in the other ear. The patients are likely to claim this symptom as binaural diplacusis.

We do not know why the latter half of the subjects heard the tones at same pitches in both ears in the second experiment. The subjects might have perceived the tone predominantly in their right ears because the overall levels of the stimuli in the left ear were lower than those in the right ear or it might be due to binaural pitch fusion, which is fusion of dichotically presented tones that evoke different pitches between the ears [9]. It is also possible that the tone presentation protocol in both ears may have been inappropriate because it resembled the protocol used to cause the octave illusion, which was discovered by Deutsch [10]. She reported a stimulus configuration that consisted of a sequence of tones, alternating in pitch between 400 Hz and 800 Hz, and was delivered to both ears simultaneously; however, when the right ear received the high tone, the left ear received the low tone, and vice versa. Instead of hearing two alternating pitches, most subjects heard a single tone that alternated between ears, while at the same time, its pitch alternated between high and low. Because interpretation of the results regarding binaural tone stimulation appears to be very complicated, we need further investigation to clarify if subjects will perceive what is equivalent to binaural diplacusis by binaural complex tone stimulation.

We believe that the above-mentioned mechanism can explain, in part, the symptoms that Meniere's disease patients experience because this disorder leads to low- to mid-frequency sensorineural hearing loss [11]. Endolymphatic hydrops plays an important part in the pathogenesis of Meniere's disease [12,13]. In addition to a human experiment that showed pitch shift with Meniere's disease [5], mechanical cochlear models of endolymphatic hydrops showed that the places of maximal displacement of the traveling waves are shifted, which must be considered the equivalent of diplacusis [14]. Our present study is not comparable to these previous studies because we used complex tones as stimulation. The previous studies have shown that pitch shift occurs when impaired ears hear a pure tone. In contrast, our study showed the presence of pitch shift with the assumption that impaired ears cannot hear the low tone, by enrolling a sample of healthy subjects. As described in this paper, pitch shift is induced by the remaining higher components when subjects hear certain complex tones. It can be induced regardless of the underlying etiology, taking into consideration that our study enrolled healthy subjects.

Because complex tones encountered in everyday life may contain higher components than those in our experiment, patients with low-tone hearing loss may not always experience bilateral diplacusis caused by the mechanism we have hypothesized here. However, sounds are subject to reflections and refractions caused by walls or objects in their path. The sound representation reaching the ear will thus differ somewhat from that initially generated. Diffraction occurs at lower frequencies because lower-frequency sounds have a longer wavelength [6]. Thus, for example, some object on the path of the sound may act as a lowpass filter after the sound bends around it, and the patients may experience diplacusis when they hear the sound without higher components. As mentioned, several conditions are required to induce binaural diplacusis by complex tone stimulation. Thus, it is unclear how closely this proposed mechanism is related to binaural diplacusis. Investigations with patients with low-tone hearing loss will be needed to clarify this issue.

## Conclusions

Under certain conditions, complex tone stimulation may induce binaural diplacusis when low-tone hearing is impaired in one ear, although there may be other mechanisms causing this phenomenon.

## Supporting information

S1 Table Stimulation tones for the first experiment. (XLSX)

S2 Table Stimulation tones for the second experiment. (XLSX)

S3 Table Subjects' responses. (XLSX)

## References

[1] Colin D, Micheyl C, Girod A, Truy E, Gallégo S. Binaural diplacusis and its relationship with hearing-threshold asymmetry. PLoS ONE 2016;11: e0159975 10.1371/journal.pone.0159975 27536884 PMC4990190

[2] Gaeth JH, Norris TW. Diplacusis in unilateral high-frequency hearing losses. J Speech Hear Res. 1965;8: 63–75. 14311806 10.1044/jshr.0801.63

[3] Ogura M, Kawase T, Kobayashi T, Suzuki Y. Modified binaural pitch-matching test for the assessment of diplacusis. Int J Audiol. 2003;42: 297–302. 14570236 10.3109/14992020309101321

[4] Paparella MM. Pathogenesis and pathophysiology of Meniere's disease. Acta Otolaryngol Suppl. 1991;485: 26–35. 1843169 10.3109/00016489109128041

[5] Brännström KJ, Grenner J. Long-term measurement of binaural intensity and pitch matches. II. Fluctuating low-frequency hearing loss. Int J Audiol. 2008;47: 675–687. 10.1080/14992020802215870 19031226

[6] Moore BCJ. An introduction to the psychology of hearing. 6th ed Leiden: Brill; 2013.

[7] Ichimiya I, Ichimiya H. Development and validation of a novel tool for assessing pitch discrimination. Auris Nasus Larynx 2016;43: 68–73. 10.1016/j.anl.2015.07.002 26277374

[8] Ladd DR, Turnbull R, Browne C, Caldwell-Harris C, Ganushchak L, Swoboda K, et al Patterns of individual differences in the perception of missing-fundamental tones. J Exp Psychol Hum Percept Perform. 2013;39: 1386–1397. 10.1037/a0031261 23398251

[9] Reiss LA, Shayman CS, Walker EP, Bennett KO, Fowler JR, Hartling CL, et al Binaural pitch fusion: Comparison of normal-hearing and hearing-impaired listeners. J Acoust Soc Am. 2017;141: 1909–1920. 10.1121/1.4978009 28372056 PMC5848869

[10] Deutsch D. An auditory illusion. Nature 1974;251: 307–309. 4427654 10.1038/251307a0

[11] Goebel JA. 2015 Equilibrium Committee Amendment to the 1995 AAO-HNS Guidelines for the Definition of Meniere's Disease. Otolaryngol Head Neck Surg. 2016;154: 403–404. 10.1177/0194599816628524 26884364

[12] Ichimiya I, Adams JC, Kimura RS. Changes in immunostaining of cochleas with experimentally induced endolymphatic hydrops. Ann Otol Rhinol Laryngol. 1994;103: 457–468. 10.1177/000348949410300607 8203812

[13] Schuknecht HF, Gulya AJ. Endolymphatic hydrops. An overview and classification. Ann Otol Rhinol Laryngol Suppl. 1983;106: 1–20. 6414357 10.1177/00034894830920s501

[14] Tonndorf J. Endolymphatic hydrops: mechanical causes of hearing loss. Arch Otorhinolaryngol. 1976;212: 293–299. 990091 10.1007/BF00453677

